# Induction of deubiquitinating enzyme USP50 during erythropoiesis and its potential role in the regulation of Ku70 stability

**DOI:** 10.1136/jim-2017-000622

**Published:** 2017-11-03

**Authors:** Junting Cai, Jianxin Wei, Valerie Schrott, Jing Zhao, Grant Bullock, Yutong Zhao

**Affiliations:** 1 Department of Medicine, University of Pittsburgh, Pittsburgh, Pennsylvania, USA; 2 Medical School, Xiangya Hospital of Central South University, Changsha, Hunan, China; 3 Department of Pathology, Vascular Medicine Institute, University of Pittsburgh, Pittsburgh, Pennsylvania, USA; 4 Vascular Medicine Institute, University of Pittsburgh, Pittsburgh, Pennsylvania, USA

## Abstract

Anemia is a very common blood disorder that affects the lives of billions of people worldwide. Anemia is caused by the loss of blood, increased destruction of red blood cells (RBCs), or reduced production of RBCs. Erythropoiesis is the complex process of RBC differentiation and maturation, in which protein degradation plays a crucial role. Protein ubiquitination regulates programmed protein degradation, which can be reversed by deubiquitinating enzymes (DUBs); however, the role of DUBs in erythropoiesis has not been well studied. We examined the expression of DUBs during erythropoiesis using an ex vivo human CD34+ hematopoietic progenitor cell culture system. Here we show that ubiquitin-specific protease 50 (USP50) levels are increased during erythropoiesis. USP50 mRNA levels are significantly increased on day 3 and protein levels are elevated on day 9 of erythroid differentiation. Coimmunoprecipitation and proteomics analyses reveal that Ku70, a DNA-binding protein, is associated with USP50. Overexpression of USP50 has no effect on Ku70 mRNA levels, while it reduces Ku70 protein levels by promoting Ku70 degradation, suggesting that USP50 may indirectly regulate Ku70 protein stability. USP50 protein is also not stable. USP50 protein degradation is independent of the proteasomal and the lysosomal degradation systems. This study suggests that DUBs like USP50 may regulate protein stability during erythropoiesis; however, more investigation is warranted.

## INTRODUCTION

Red blood cells (RBCs) are the most abundant cells in our circulatory system. RBCs are highly specialized to transport oxygen, bound to intracellular hemoglobin, from the lungs to the rest of the body. RBCs are responsible for maintaining normal tissue oxygenation and preventing systemic and cellular hypoxia. Anemia is a very common disorder that results from decreased numbers of circulating RBCs or a decreased capacity of RBCs to carry oxygen. The symptoms felt by patients with anemia are related to systemic hypoxia. Conditions such as blood loss, hemolysis, iron deficiency, vitamin deficiencies, and chronic inflammation are common causes of anemia.^1,2^

The lifespan of circulating RBCs is 90–120 days, and under basal conditions approximately 1×10^6^ damaged or senescent RBCs are removed from the circulation every second. To keep up with RBC turnover and maintain tissue normoxia, RBC production proceeds at an incredibly rapid rate in the bone marrow of healthy humans. Erythropoiesis is the highly programmed process of producing mature RBCs from hematopoietic stem cells. Erythropoiesis proceeds in a stepwise manner over several days and involves many morphologically and molecularly distinct stages. Erythropoiesis depends on an array of transcription factors and cytokines that orchestrate a regulated cascade of replication, differentiation, apoptosis, chromatin condensation, enucleation, mitophagy, and cytoskeletal maturation.^3,4^

In the final stages of RBC maturation, the nucleus, endoplasmic reticulum, and mitochondria must be removed or degraded.^5–7^ The regulated degradation of protein plays a critical role in these final stages of erythropoiesis. Likewise, protein degradation plays an essential role in the chromatin remodeling that occurs during the earliest lineage commitment steps of erythropoiesis.^8^ Selective protein degradation contributes to RBC differentiation.^6,9,10^ Protein degradation and stability are regulated by ubiquitin post-translational modification. Ubiquitin is an 8 kDa protein that marks proteins for degradation in the proteasome or lysosome. Ubiquitination is catalyzed by the sequential action of three classes of enzymes: ubiquitin-activating enzyme (E1), ubiquitin-conjugating enzyme (E2), and ubiquitin ligase (E3).^11,12^ Ubiquitination plays an important role in erythropoiesis. Wefes *et al*^10^ revealed that the mRNA for two E2s (E2-20K and E2-230K) increased, while mRNA for two other E2s was reduced during the terminal stage of erythropoiesis. Nguyen *et al*^13^ showed that an inactivating mutation in murine E2-230K (also called Ube20) resulted in anemia. Trim58, a ubiquitin E3 ligase, degrades dynein and promotes enucleation during terminal erythropoiesis.^14^ Protein ubiquitination and degradation are highly active during erythropoiesis, but their regulation is obscure.

Ubiquitination is reversible by deubiquitinating enzymes (DUBs). DUBs remove the ubiquitin monomers or multimers from protein substrates, thus improving their stability. So far, approximately 100 DUBs have been identified and cloned. DUBs are involved in cell proliferation, growth, mobility, death, and differentiation.^15^ However, very little is known about the role of DUBs in erythropoiesis. Here, we show in primary human erythroid progenitor cells that several DUBs transcripts, including ubiquitin-specific protease 50 (USP50), are increased during erythropoiesis. USP50 has been shown to regulate cell cycle and DNA repair through interaction with the Wee1 protein in transformed human osteosarcoma and human epithelial cell lines.^16,17^ USP50 has also been shown to play a role in inflammasomal function through targeting the adaptor protein.^16,17^ In this study, our results suggest that USP50 indirectly regulates the stability of the Ku70, which is a DNA-binding protein, and is expressed in CD34+ hematopoietic progenitor cells (HPCs).^18^ This is the first direct biochemical investigation of DUBs in erythropoiesis and the first time that USP50 has been implicated in erythropoiesis.

## MATERIALS AND METHODS

### Cell cultures and reagents

Granulocyte colony stimulating factor (G-CSF)-mobilized, CD34+ human HPCs were harvested from peripheral blood of healthy donors and de-identified according to an institutional review board- approved protocol by the Hematopoietic Cell Processing Core of the Fred Hutchinson Cancer Research Center (Seattle, Washington, USA). The HPCs were first expanded in Serum-Free Expansion Medium (StemSpan SFEM, STEMCELL Technologies, Cambridge, Massachusetts, USA), supplemented with 1× cytokine cocktail 100 (CC100, STEMCELL Technologies), 1 µg/mL hydrocortisone (Sigma), and 1.5 U/mL epoetin alfa (Procrit, Amgen, Thousand Oaks, California, USA). After 5 days of expansion, the cells were harvested, washed, and placed into differentiation medium that comprised StemSpan SFEM, 4.5 U/mL Procrit, and 10 ng/mL recombinant human stem cell factor (PeproTech, Rocky Hill, New Jersey, USA). All cultures were maintained at 37°C and 5% CO_2_ in a humidified cell culture incubator. Cells were harvested on differentiation days 0, 1, 3, 5, and 9.

HEK293T cell line (ATCC, Manassas, Virginia, USA) was cultured in Dulbecco’s Modified Eagle’s Medium supplemented with 10% fetal bovine serum (FBS), 1% non-essential amino acids, 1% L-glutamine, and 1% sodium pyruvate. Mouse lung epithelial (MLE12, ATCC) cells were cultured in hydrocortisone, insulin, transferrin, estrogen and selenium (HITES) medium supplemented with 10% FBS. Cycloheximide (CHX), leupeptin, and antibody against β-actin were purchased from Sigma-Aldrich (Cambridge, Massachusetts, USA). MG132 was purchased from EMD Millipore (Billerica, Massachusetts, USA). V5 antibody and the mammalian expression plasmid pcDNA3.1/V5-His TOPO, *Escherichia coli* Top 10 competent cells and Lipofectamine were purchased from Life Technologies (Carlsbad, California, USA). Ku70 antibody was from Cell Signaling (Danvers, Massachusetts, USA). Antibody against USP50 was from Proteintech (Rosemont, Illinois, USA).

### RNA extraction and qPCR

Total RNA was extracted from HPCs or from MLE cells according to the manufacturer’s instructions (Bio-Rad, Hercules, California, USA). After isolation, RNA was immediately reversely transcripted to cDNA using a cDNA synthesis kit (Bio-Rad). Quantitative polymerase chain reaction (qPCR) was performed using iQ SYBR Green Supermix and the iCycler Real-Time PCR Detection System (Bio-Rad). Target amplicon expression signals in each sample were normalized to the signal for *Gapdh*.

### Western blotting

Cells were washed with phosphate buffered saline (PBS) and collected in lysis buffer (20 mM Tris-HCl, 150 mM NaCl, 2 mM ethylene glycerol Tetraacetic Acid (EGTA) 5 mM β-glycerophosphate, 1 mM MgCl_2_, 1% Triton X-100, 1 mM sodium orthovanadate, 10 µg/mL of protease inhibitors, 1 µg/mL aprotinin, 1 µg/mL leupeptin, and 1 µg/mL pepstatin). Protein concentrations of cell lysates were determined using a Bradford assay (Bio-Rad). Twenty micrograms of whole cell lysates were subjected to 4%–15% SDS-PAGE, then transferred to nitrocellulose membranes. Membranes were blocked with 5% non-fat milk in TBST (25 mM Tris-HCl, pH 7.4, 137 mM NaCl, and 0.1% Tween 20) for 1 hour, and incubated with primary antibodies in 5%=Bovine Serum Albumin (BSA) in TBST overnight, washed and followed by incubation with secondary antibodies. The membranes were developed with a chemiluminescence detection system.

### Transfection

MLE12 cells were suspended in 120 µL of 20 mM HEPES (4-(2-hydroxyethyl)-piperazineethanesulfonic acid) in PBS and mixed with USP50-V5 plasmid in a cuvette. Cells were nucleofected using the Lonna electroporation transfection system. Immediately following nucleofection, 1 mL of 10% HITES medium was added to the cuvette and the cells were plated in a 6-well plate. HEK293T cells were transfected with USP50-V5 plasmid using Lipofectamine transfection reagent. Briefly, USP50-V5 plasmid was incubated with Lipofectamine (1:3) in Opti-MEM medium for 5 min at room temperature. The transfection mix was added into cell culture for 24 hours.

### Coimmunoprecipitation and protein identification by LC-MS/MS

After transfection with USP50-V5 plasmid for 24 hours, HEK293T cell lysates were precleaned with protein A/G agarose, and then removed from the protein-A/G agarose and incubated with 5 µg/mL anti-V5 antibody at 4°C. After overnight incubation, 40 µL of protein A/G agarose beads (Santa Cruz, California, USA) were added and incubated for an additional 2 hours at 4°C with mixing on a rotator. Protein-A/G agarose beads were collected by centrifugation at 1000 g at 4°C for 1 min and washed five times with PBS, followed by separation with 10% sodium dodecyl sulfate polyacrylamide gel electrophoresis (SDS-PAGE). The acrylamide gels were cut into seven fractions and were subjected to liquid chromatography tandem-mass spectrometry (LC-MS/MS) at the BioMS Center of the University of Pittsburgh.

### Statistical analysis

All results were subjected to statistical analysis using two-way analysis of variance and, wherever appropriate, Student’s t-test. Data are expressed as mean±SD of triplicate samples from at least three independent experiments, and P values <0.05 were considered statistically significant.

## RESULTS

### USP50 is increased during erythropoiesis

**Figure 1:**
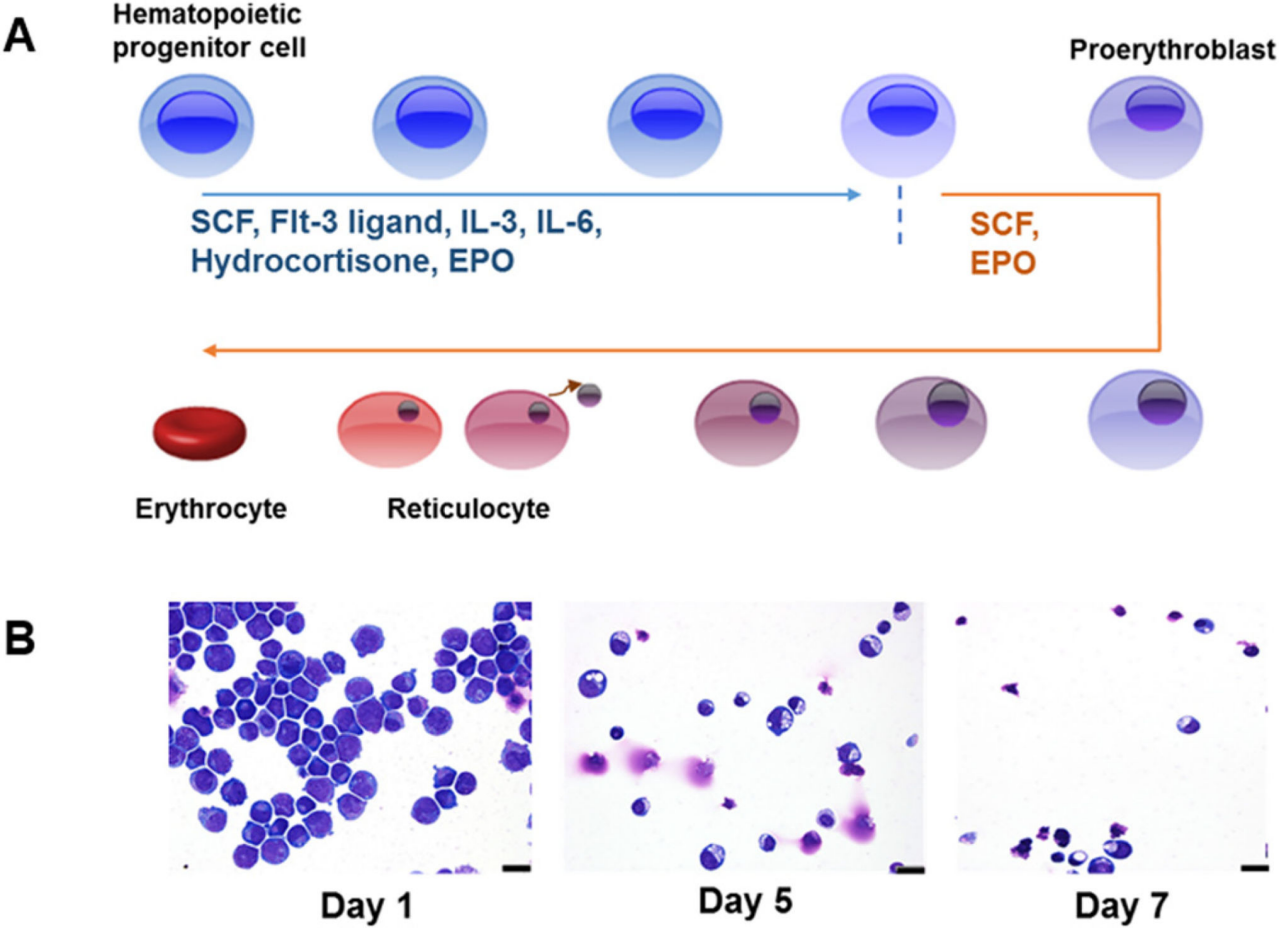
Ex vivo CD34+ human hematopoietic progenitor cell culture model of erythropoiesis. (A) Illustration of the morphological changes that occur during erythropoiesis. (B) Representative images of cells from days 1, 5, and 7 of erythroid differentiation. Cells were cytospun, fixed, and stained with the May-Grunwald and Giemsa stains. Bar, 15 nm. IL, interleukin; SCF, stem cell factor; EPO, erythropoietin.

Ubiquitination-regulated protein degradation plays a critical role in the process of RBC maturation.^6,10,13,14^ DUBs preserve protein levels by removing ubiquitin monomers or multimers from ubiquitinated proteins.^15^ However, the role of DUBs in erythropoiesis is unknown. During erythropoiesis, hematopoietic stem cells develop into mature RBCs. This process involves a series of changes in cellular morphology illustrated in figure 1A. The nucleus shrinks and the chromatin becomes more condensed as the erythroid progenitors mature. In the terminal phases of erythropoiesis, the nucleus is extruded to form reticulocytes. The endoplasmic reticulum and mitochondria are degraded in the newly formed reticulocyte as it transitions to a mature RBC. As the nucleus shrinks the cytoplasm transitions from dark blue to pink as mRNA is translated into protein. Most of the proteins in the reticulocytes are hemoglobin. During reticulocyte maturation the membrane cytoskeleton matures, producing the biconcave disc shape of a mature RBC. Figure 1B illustrates cellular morphology on days 1, 5, and 7 of erythroid differentiation. Day 1 represents the day that the cells were switched from expansion to differentiation medium.

**Figure 2:**
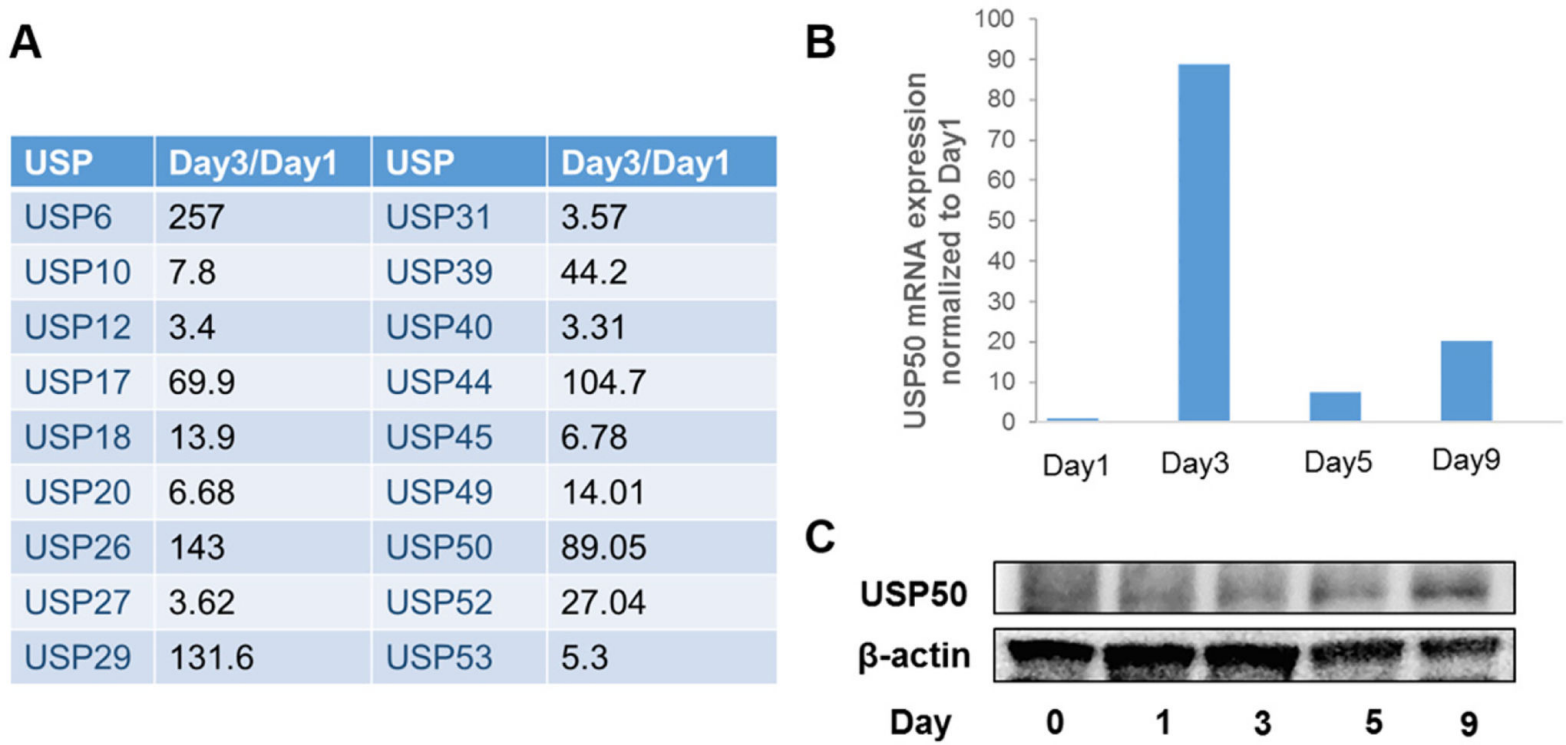
Ubiquitin-specific protease 50 (USP50) is increased during erythropoiesis. (A) Total RNA was extracted from human CD34+ hematopoietic progenitor cells (HPCs) on day 1 and day 3 of erythroid differentiation. Real-time quantitative PCR was used to compare ubiquitin-specific protease mRNA levels. Amplification of each sample was normalized to its glyceraldehyde-3-phosphate dehydrogenase (GAPDH) transcript levels. Shown are the ratios of day 3/day 1 in a representative experiment from five independent experiments. (B) Total RNAs were extracted on days 1, 3, 5, and 9 from HPCs. USP50 mRNA levels were measured by real-time qPCR. Shown are data in a representative experiment from five independent experiments. (C) Whole cell lysates were made from HPCs on days 0, 1, 3, 5, and 9 of erythroid differentiation. USP50 and β-actin proteins were detected by immunoblot. Representative blots were shown from five independent experiments.

RNA was collected from the ex vivo erythropoiesis cultures on day 1 and day 3. Ubiquitin-specific protease gene expression was examined by real-time qPCR. Figure 2A shows that mRNA levels of USP6, 17, 26, 29, 39, 44, 50, and 52 were dramatically increased on day 3 of erythroid differentiation, compared with their levels on day 1 of differentiation. USP50 has not been well characterized; however, a recent study indicated that USP50 regulated DNA integrity by stabilization of Wee1, a protein kinase in the regulation of cell cycle.^16^ Previous studies demonstrate that ubiquitination plays a critical role in the enucleation that occurs late in erythropoiesis. USP50 mRNA levels were elevated on day 3, and reduced on day 5 and day 9 (figure 2A). A decrease in USP50 during days 5–9 correlates with increased enucleation observed during these days in the ex vivo culture system shown in figure 1. Figure 2B shows that USP50 protein levels were barely detected from day 1 to day 3 of erythroid differentiation, but were increased on day 5 and day 9.

### Ku70 is a USP50-associated protein

**Figure 3:**
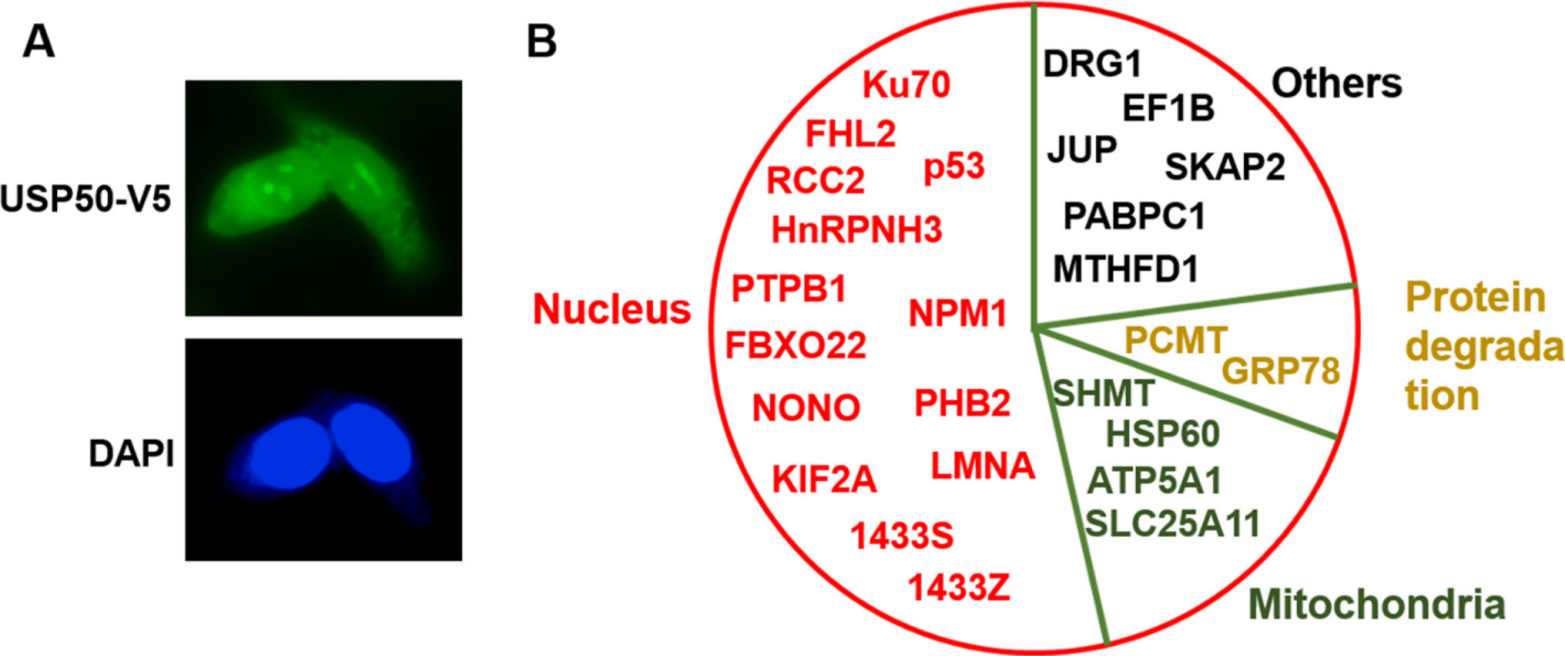
Ubiquitin-specific protease 50 (USP50) is localized in the nuclei and its binding partners are identified. (A) Mouse lung epithelial (MLE12) cells were transfected with USP50-V5 plasmids and incubated for 24 hours. Cells were fixed and stained with a V5 antibody. USP50-V5: green; DAPI (4',6'-diamidino-2-phenylindole, nuclear DNA stain): blue. (B) HEK293T cells were transfected with USP50-V5 plasmid and incubated for 24 hours. Immunoprecipitation with a V5 antibody, followed by SDS-PAGE and LC-MS/MS analysis. Shown are potential USP50 binding partners separated by known intracellular location or function based on literature.

In order to play a role in enucleation, USP50 should localize to the nucleus or perinucleus. Thus, we examined the cellular localization of overexpressed V5-antigen-tagged USP50 in MLE12 cells, since this cell line has high plasmid transfection efficiency and is suitable for immunostaining. Figure 3A shows that USP50-V5 localizes to both the cytoplasm and nuclei. Coimmunoprecipitation experiments followed by LC-MS/MS analysis demonstrates that USP50 potentially binds to 26 candidate proteins in HEK293T cells, a cell line often used for highly overexpression studies (figure 3B). Over half (53%) of these potential USP50 interacting proteins are nuclear or nuclear-related proteins, such as Ku70, HnRPNH3, and FHL2. Four mitochondrial proteins were also identified in this screen for USP50-binding proteins. This suggests that USP50 may play a role in mitochondrial protein stability or localization. Ku70 is found in most human cell types and tissues, but the amount Ku70 protein and its intracellular localization is variable.^19^ Ku70 binds to double-stranded breaks in DNA and has been shown to play a role in the repair of DNA damage. Interestingly, Ku70 has been implicated in the deubiquitination and stabilization of Mcl-1, which is an antiapoptotic member of the BCL-2 protein family.^20^ Mcl-1 plays a critical role as a suppressor of apoptosis during erythropoiesis.^21^ These observations suggest that the interaction of Ku70 with USP50 may play a critical role in stabilizing Mcl-1 and controlling apoptosis during erythropoiesis.

### USP50 reduces Ku70 protein stability without affecting its mRNA levels

**Figure 4:**
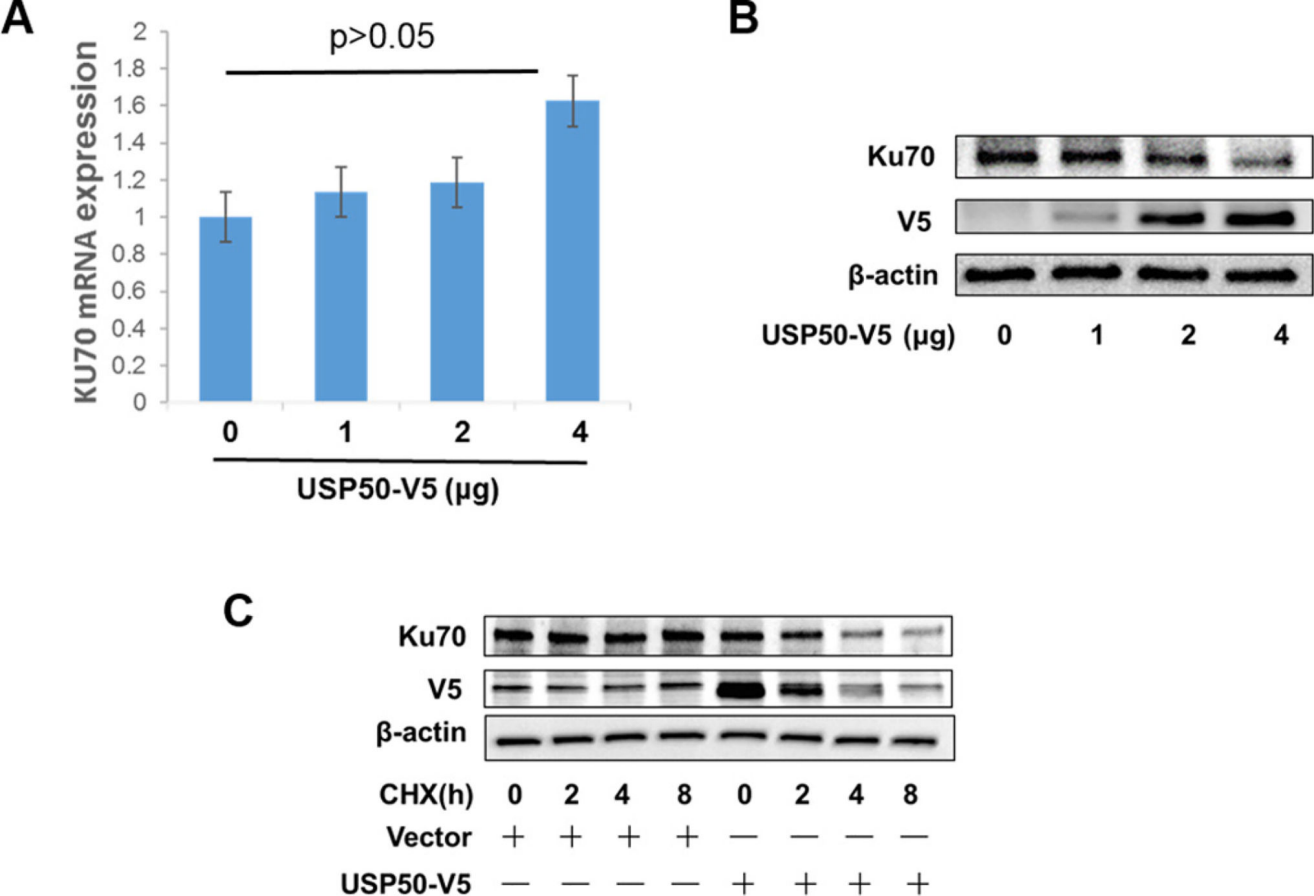
Ubiquitin-specific protease 50 (USP50) reduces Ku70 protein stability. (A) Mouse lung epithelial (MLE12) cells were transfected with 0–4 µg USP50-V5 plasmids per 35 mm dish and incubated for 24 hours. Total RNA was extracted and Ku70 mRNA levels were compared by real-time qPCR. The expression of Ku70 transcripts was normalized to *Gapdh*. The results were also normalized to 0 µg of USP50 plasmid. (B) MLE12 cells were transfected with USP50-V5 plasmids (0–4 µg of DNA per 35 mm dish) and incubated for 24 hours. Protein expression in whole cell lysates was analyzed by immunoblotting with antibodies against Ku70, V5, and β-actin. (C) MLE12 cells were transfected with USP50-V5 plasmid and incubated for 24 hours. Cells were treated with cycloheximide (CHX) (40 µg/mL) for 0–8 hours. Expression of proteins in whole cell lysates was analyzed by immunoblotting with antibodies against Ku70, V5, and β-actin.

To determine whether USP50 affects Ku70 expression, Ku70 mRNA levels were evaluated in USP50-V5 overexpressing MLE12 cells. As shown in figure 4A, overexpression of USP50 had no significant effect on Ku70 transcripts. Figure 4B shows that overexpression of USP50-V5 protein reduced Ku70 protein in a dose-dependent manner based on immunoblot. USP50 had no effect on Ku70 mRNA levels, and this result suggests that USP50 might regulate Ku70 protein stability. Cells were treated with 40 µg/mL CHX to inhibit de novo protein synthesis, and cell lysates were made every 2 hours for a total of 8 hours (figure 4C). The results from this CHX chase assay (figure 4C) showed that Ku70 protein levels did not change after CHX treatment for 8 hours. Ku70 exhibits a long half-life of greater than 8 hours. However, in USP50-V5 overexpressing cells, the half-life of Ku70 is reduced to approximately 4 hours (figure 4C). These data suggest that USP50 reduces Ku70 protein stability. This is an unusual function for a DUB, but it is possible that USP50 stabilizes an unknown E3 ligase that targets Ku70 for degradation. Figure 3B shows that USP50 is associated with the FBXO22, a subunit of a nuclear ubiquitin E3 ligase. Future studies will investigate USP50–FBXO22–Ku70 interactions.

### USP50 is not a stable protein

**Figure 5:**
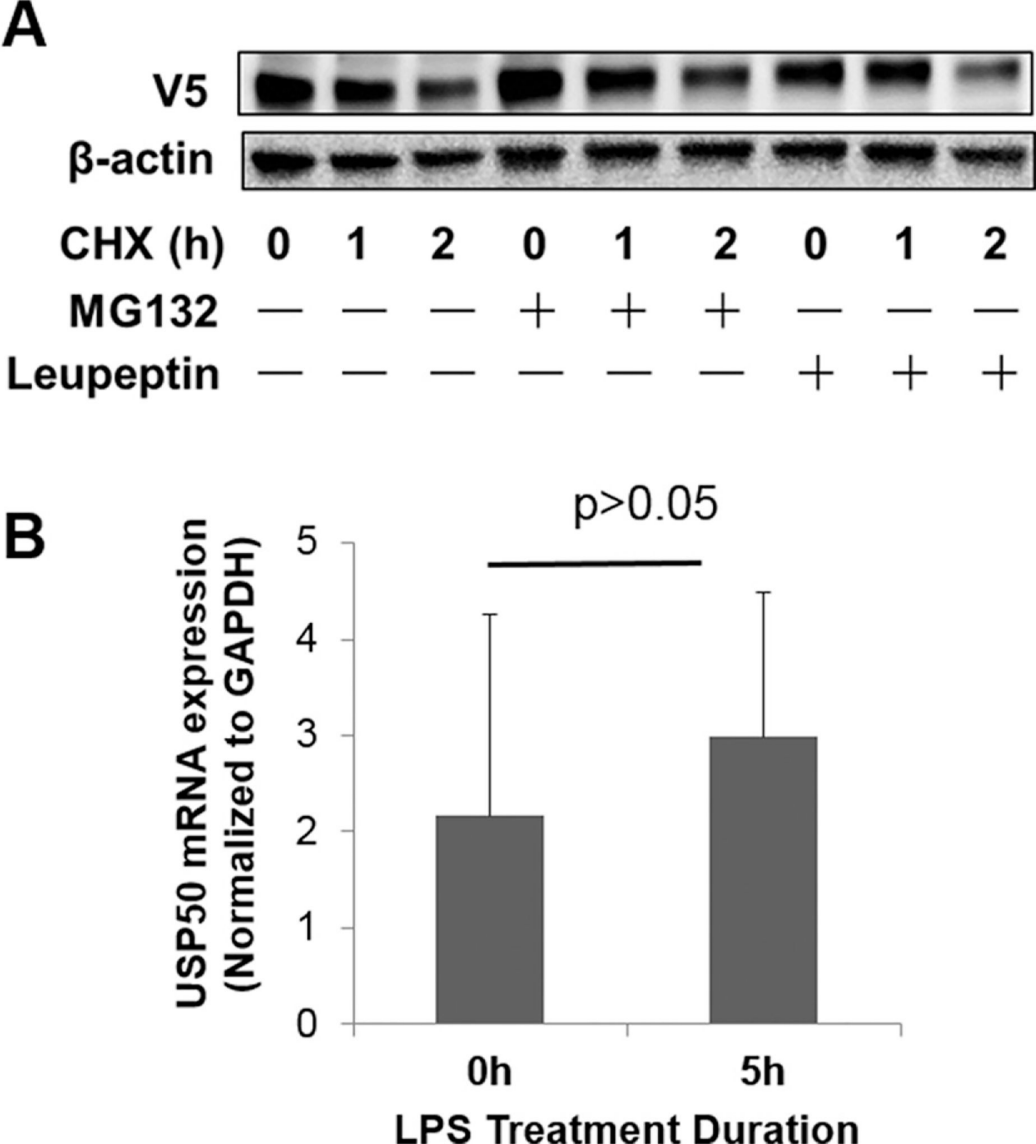
Ubiquitin-specific protease 50 (USP50) is a not stable protein. (A) Mouse lung epithelial (MLE12) cells were transfected with USP50-V5 plasmids and incubated for 24 hours. Cells were treated with cycloheximide (CHX) (40 µg/mL) with or without MG132 (20 µg/mL) or leupeptin (20 µg/mL) for 0–2 hours. Cell lysates were analyzed by immunoblotting with antibodies against V5 and β-actin. (B) MLE12 cells were transfected with USP50-V5 and treated with 5 µg/mL lipopolysaccharide (LPS) for 5 hours. Total RNA was extracted and USP50 mRNA levels were determined by real-time qPCR. The expression of USP50 transcripts was normalized to *Gapdh*.

Figure 4C also shows that V5-tagged USP50 protein has a short half-life of approximately 2 hours. Protein degradation is commonly mediated by the proteasomal or lysosomal systems. USP50-V5 overexpressing cells were treated with CHX and either a proteasome inhibitor (MG132) or a lysosome inhibitor (leupeptin). As shown in figure 5A, USP50-V5 protein levels were reduced to approximately 50% of untreated control cells after 2 hours of CHX. Figure 5A also shows that neither MG132 nor leupeptin extended the half-life of USP50-V5 protein. These data suggest that USP50-V5 degradation is not mediated by the proteasome or the lysosome.

USP50 plays a role in inflammatory responses,^17^ but it is unclear if inflammatory signaling alters USP50 expression. Figure 5B shows that lipopolysaccharide has no effect on USP50 mRNA levels.

## DISCUSSION

Erythropoiesis is a complex process that includes dramatic cellular transitions from nucleated stem and progenitor cells to enucleated reticulocytes and RBCs.^3,5^ Defects in erythropoiesis result in anemia. Selective regulation of protein ubiquitination and deubiquitination plays a critical role in erythropoiesis by guiding the removal of proteins and organelles through the proteasome or lysosome.^11,12^ DUBs stabilize proteins by deubiquitinating them and preventing their degradation; however, little is known about the role of DUBs in erythropoiesis.^15^ This study shows that the DUB, USP50, is upregulated during the late stages of erythropoiesis. USP50 is localized to both the cytoplasm and nucleus, and it exhibits a short half-life. Interestingly, USP50 associates with the Ku70 protein and reduces the half-life of Ku70 protein. Ku70 is a DNA-binding protein that has been associated with deubiquitinating activity in prior studies.^20^ The antiapoptotic BCL2 family member, Mcl-1, is a target of Ku70-mediated deubiquitination.^20^ Mcl-1 plays an important role in erythropoiesis by regulating apoptotic rate.^21,22^ The current study is the first to reveal an upregulation of USP50 during erythropoiesis. This is also the first study to demonstrate an interaction between USP50 and Ku70. Taken together, these findings suggest a model where USP50 and Ku70 may stabilize Mcl-1 and prevent apoptosis in the late stages of erythropoiesis. Future experiments will investigate the effects of USP50 and Ku70 on Mcl-1 stability in late-stage erythroblasts.

Cell cycle arrest and exit from the cell cycle are required during or just before enucleation in terminal erythropoiesis. Li *et al*^9^ showed that Cul4A, a subunit of a ubiquitin ligase, regulates cell cycle exit by targeting p27. Endogenous Cul4A expression is decreased during the differentiation. The role of USP50 in cell cycle exit during terminal erythropoiesis is unknown. USP50 has also been identified as a cell cycle regulator by stabilizing Wee1, a cell cycle inhibitor kinase.^16^ Aressy *et al*^16^ revealed a crucial role of USP50 in the regulation of cell cycle, especially the G2/M checkpoint. In the current study, USP50 is increased during the late stages of erythropoiesis, suggesting that USP50 may play a role in cell cycle arrest during terminal erythropoiesis.

Coimmunoprecipitation experiments combined with proteomics analysis identified several potential USP50 interacting proteins or substrates. The Ku70 forms a heterodimer with Ku80, which binds to chromosomal replication origins^23^ and plays a critical role in cell cycle and DNA replication.^24^ The current study reveals that USP50 is associated with Ku70 and overexpression of USP50 reduces Ku70 protein stability. DUBs, in general, stabilizes substrates. Thus, we hypothesize that USP50-mediated Ku70 degradation might be through targeting a ubiquitin E3 ligase that ubiquitinates and degrades Ku70. Cytosolic Ku70 is known to be ubiquitinated by a ubiquitin E3 ligase, Hdm2^25^; however, the nuclear Ku70 ubiquitination has not been reported. Interestingly, we found that a nuclear ubiquitin E3 ligase, FBXO22, is in the protein complex of USP50 and Ku70. Future studies will be focused on investigating whether USP50 stabilizes FBXO22, which ubiquitinates and degrades Ku70. The discovery of USP50/Ku70 link in this study provides a further evidence that USP50 may play a role in the regulation cell cycle exit during terminal erythropoiesis.

USP50 expression is upregulated during terminal erythropoiesis, and this suggests a role for DUBs in cell cycle exit or enucleation during terminal erythropoiesis. These studies are limited and raise many questions about how USP50 and Ku70 may regulate terminal stages of erythropoiesis. Generation of USP50 knockout mice may be useful to understand the effects of USP50 on cell cycle or enucleation during erythropoiesis. Additional studies are necessary to elucidate the molecular mechanisms of USP50-mediated destabilization of Ku70 protein. Nevertheless, this is the first report to investigate the role of DUBs in erythropoiesis.
